# α-Linolenic Acid-Enriched Cold-Pressed Perilla Oil Suppress High-Fat Diet-Induced Hepatic Steatosis through Amelioration of the ER Stress-Mediated Autophagy

**DOI:** 10.3390/molecules25112662

**Published:** 2020-06-08

**Authors:** Su Ji Bae, Ji Eun Kim, Hyeon Jun Choi, Yun Ju Choi, Su Jin Lee, Jeong Eun Gong, Sungbaek Seo, Seung Yun Yang, Beum-Soo An, Hee Seob Lee, Dong Seob Kim, Chung Yeoul Lee, Dae Youn Hwang

**Affiliations:** 1 Department of Biomaterials Science, College of Natural Resources and Life Science/Laboratory Animal Resources Center, Pusan National University, Miryang 50463, Korea; sujibaebae@pusan.ac.kr (S.J.B.); prettyjiunx@naver.com (J.E.K.); rudwns546@naver.com (H.J.C.); poiu335@naver.com (Y.J.C.); nuit4510@naver.com (S.J.L.); kos93589@naver.com (J.E.G.); sbseo81@pusan.ac.kr (S.S.); syang@pusan.ac.kr (S.Y.Y.); anbs@pusan.ac.kr (B.-S.A.); 2 Department of Food Science and Nutrition, College of Human Ecology, Pusan National University, Busan 46241, Korea; heeseoblee@pusan.ac.kr; 3 Department of Food Engineering, College of Natural Resources and Life Science, Pusan National University, Miryang 50463, Korea; kds@pusan.ac.kr; 4 Gangrim Organics, Miryang 50463, Korea; lcy9@hanmail.net

**Keywords:** obesity, cold-pressed perilla oil, linolenic acid, hepatic steatosis, ER stress, autophagy

## Abstract

Perilla oil has been considered to have excellent potential for treating various diseases due to its contents of beneficial fatty acids, such as α-linolenic acid, oleic acid and linoleic acid. The therapeutic effects and molecular mechanism of an α-linolenic acid-enriched cold-pressed perilla oil (LEP) on hepatic steatosis of an obesity model were investigated by analyzing alterations in fat accumulation and endoplasmic reticulum (ER) stress-mediated autophagy, in high-fat diet (HFD)-induced obesity C57BL/6N mice treated with LEP for 16 weeks. Although no significant alterations were detected in body weight and most organ weights, the liver weight and accumulation of lipid droplets in the liver section were significantly lower in HFD + LEP treated group as compared to the HFD + Vehicle treated group. Reduced mRNA expression levels of adipogenesis and lipogenesis regulating factors, including the peroxisome proliferator-activated receptor (PPAR)_γ_, CCAAT/enhancer-binding protein (C/EBP)α, fatty acid synthase (FAS), and adipocyte fatty acid-binding protein 2 (aP2) were observed after LEP treatment for 16 weeks, while the levels of lipolysis were remarkably increased in the same group. Moreover, the LEP-treated groups showed suppression of ER stress-regulating factors, such as the C/EBP homologous protein (CHOP), eukaryotic translation initiation factor 2α (eIF2α), inositol-requiring protein 1 (IRE1)α, and Jun-N-terminal kinase (JNK) during anti-hepatic steatosis effects. The expression level of the microtubule-associated protein 1A/1B-light chain 3 (LC3) protein and phosphatidylinositol-3-kinase (PI3K)/AKT/ mammalian target of rapamycin (mTOR) pathway for the autophagy response showed a significant decrease in the HFD+LEP-treated group. Furthermore, ER stress-mediated autophagy was accompanied with enhanced phosphorylation of extracellular signal-regulated kinase (ERK), JNK, and p38 protein in the mitogen-activated protein (MAP) kinase signaling pathway. Taken together, the results of the present study indicate that treatment with LEP inhibits hepatic steatosis in the HFD-induced obese model through regulation of adipogenesis and lipolysis. We believe our results are the first to show that the anti-hepatic steatosis activity of α-linolenic acid from cold-pressed perilla oil might be tightly correlated with the amelioration of ER stress-mediated autophagy.

## 1. Introduction

One of nonalcoholic fatty liver disease (NAFLD) is accompanied by intracellular accumulation of lipid droplets in the liver as hepatic steatosis [1]. This disease effectively progresses to various liver disorders, including nonalcoholic steatohepatitis (NASH), fibrosis, cirrhosis, and hepatocellular carcinoma [1,2]. At the same time, hepatic steatosis increases the risk for the development of type II diabetes, insulin resistance, hypertension, and obesity-related mortality [3,4]. Several previous studies on steatosis in the liver tissue of NAFLD, genetic, and diet-induced obesity (DIO) models show scientific evidence correlating hepatic steatosis and ER stress [5,6]. ER stress-mediated hepatic steatosis and lipogenesis were especially detected in the tunicamycin injected model, ob/ob mice, and the HFD-induced model [6,7,8,9,10]. A positive feedback loop between ER stress and hepatic steatosis is considered as one of the causes for serious liver injury [11]. Also, autophagy is a well-known lysosomal degradative pathway that stimulates cell survival through the regulation of energy supplements or defective organelle elimination during injury conditions [12]. This pathway is linked to the reduction of intracellular lipid droplets, attenuation of inflammation, and improvement of hepatic injury in NAFLD [13]. However, the role and mechanism of ER stress-mediated autophagy in hepatic steatosis remains unclear, although many studies have shown evidence for the interplay between ER stress and inflammation in the pathogenesis of hepatic steatosis [14,15].

Perilla seeds and its oil have garnered great attention for providing health benefits due to their various medicinal properties and phytochemical contents. Perilla seeds are rich in numerous phenolic compounds, including rosmarinic acid, rosmarinic acid-3-O- glucoside, caffeic acid, ferulic acid, and caffeic acid-3-O-glucoside, as well as several flavonoids such as apigenin, luteolin, and catechin [16]. However, the perilla oil extracted from seeds majorly consist of α-linolenic acid (C18:3), linoleic acid (C18:2), palmitic acid (C16:1), oleic acid (C18:1), and stearic acid (C18:0). Among these, α-linolenic acid is a polyunsaturated fatty acid (PUFA) and considered as one member of omega-3 fatty acid group that have first double bonds between the third and fourth carbon [17,18]. This compound was firstly isolated from linseed oil in 1844 and the exact structure was verified in 1939 [18,19]. Also, α-linolenic acid majorly contributes to the reduction of inflammation and the prevention of some chronic diseases through the the inhibition of prostaglandin synthesis [20]. Based on the bioactive components of perilla oil, many significant pharmacological effects were investigated in cancer, diabetes, asthma, microbial infectious disease, inflammation, oxidative stress-related disease, constipation, cognitive impairment, and cardiovascular disease [21,22,23,24]. The anti-obesity effects of perilla oil were also verified in some obesity animal models. It was reported that the treatment with perilla oil containing 58% α-linolenic acid decreased the serum level of triglycerides (TG) and the weight of white adipose tissue, without altering the food intake and bodyweight of animals [25]. Administration of perilla oil-rich diet for 16 weeks ameliorated the HFD-induced hepatic steatosis and prevented an increase of TG, total cholesterol (TC), and low-density lipoprotein-cholesterol (LDL-C) levels in serum [26,27]. A similar anti-obesity effect was detected in the HFD-induced obesity model treated with 200 mg/kg and 1000 mg/kg of perilla oil for 12 weeks [28]. However, no studies provide any scientific evidence of the beneficial effects of LEP on ER stress-mediated autophagy during anti-hepatic steatosis effects, even though the outcome of the LEP-enriched western-style diet was focused on the inhibition of the lipid profile and hepatic steatosis [27].

This study was, therefore, undertaken to investigate the therapeutic responses and mechanisms during the anti-hepatic steatosis effects of LEP in an HFD-induced obesity model, and evaluate the possibility of developing cold-pressed perilla oil as a new natural medicine. Our results provide the first scientific evidence in the obesity model, that the inhibitory effects of α-linolenic acid on hepatic steatosis are associated with ER stress-mediated autophagy.

## 2. Results

### 2.1. Determination of α-Linolenic Acid Concentration in LEP

**Figure 1:**
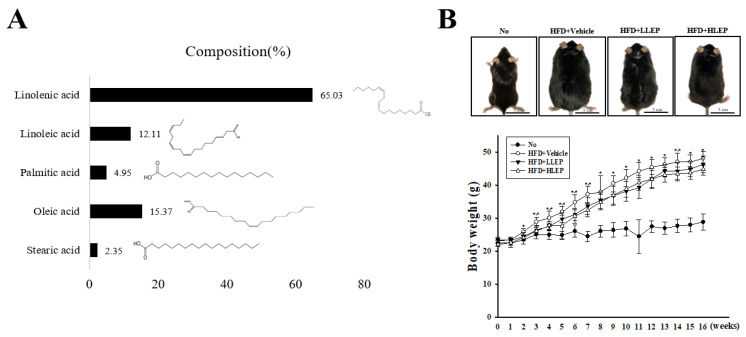
Composition of LEP and assessment of bodyweight. (**A**) GC-MS analysis of LEP contents revealed five fatty acids—linoleic acid, α-linolenic acid, stearic acid, oleic acid, and palmitic acid. (**B**) The bodyweight of No, HFD + Vehicle, HFD + LLEP-, and HFD + HLEP-treated groups were measured from 0 to 16 weeks, using a chemical balance. Five to seven mice per group were used in the bodyweight measurements. Data represent the mean ± SD. * *p* < 0.05 compared to the No treated group. # *p* < 0.05 compared to the HFD + Vehicle-treated group. Abbreviations: HFD, high-fat diet; LLEP, low concentration of LEP; HLEP, high concentration of LEP.

The LEP composition was analyzed using Gas Chromatography-Mass Spectrometry (GC-MS). Five major fatty acids were detected in LEP, including α-linolenic acid, oleic acid, linoleic acid, palmitic acid, and stearic acid. The highest concentration of fatty acid was determined to be α-linolenic acid (65.03%), followed by oleic acid (15.37%), linoleic acid (12.11%), palmitic acid (4.95%), and stearic acid (2.35%) (Figure 1A). These results indicate the potential of LEP for treating HFD-induced hepatic steatosis.

### 2.2. Suppressive Effects of LEP on Bodyweight and Serum Lipid Profile of HFD-Induced Obesity Mice

We measured the alterations in bodyweight, and serum lipid profile of HFD + LEP-treated mice to investigate the suppressive effects of LEP against obesity. As presented in Figure 1B, the bodyweights remarkably increased in the HFD-feeding group as compared to the No group. However, decreased bodyweights were detected in the HFD + LEP treated groups, although there was no significance at the several time points evaluated. A similar decrease was observed in the lipid profile factors of the HFD + LEP treated groups. Especially, the serum concentrations of TC, LDL-C, and glucose (GLU) were lower in the HFD + LEP-treated groups than the HFD + Vehicle treated group, although other factors evaluated remained constant (Table 1). The results of the present study suggest that LEP treatment for 16 weeks suppresses the bodyweight gain, as well as increases the concentrations of TC, LDL-C, and GLU in serum.

### 2.3. Inhibitory Effect of LEP on Fat Accumulation in Abdominal Fat Tissue

**Figure 2:**
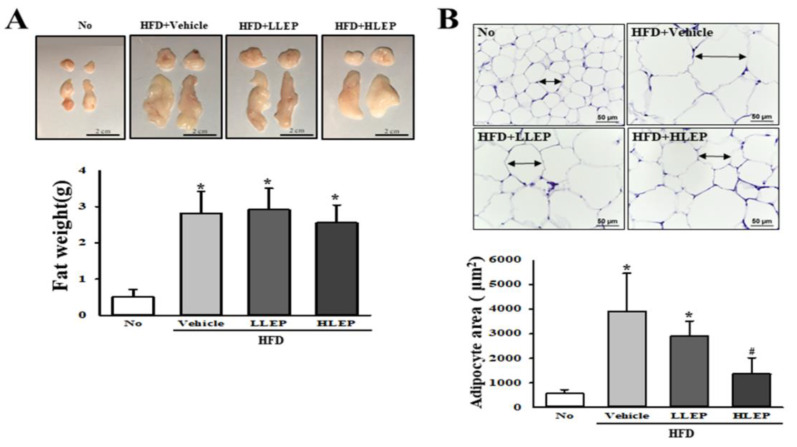
Measurement of fat weight and average area of adipocytes. (**A**) The fat weight is presented by combining the weight of the epididymis and retroperitoneal fat. In the fat image, the upper part indicates the retroperitoneal fat, and the lower part indicates the epididymis fat harvested from the abdominal region of mice of all subset groups. Five to six mice per group were used for the collection of fat tissues; the weight of fat tissue was measured in duplicate for each tissue. (**B**) After taking a photo of the fat tissue at 200× magnification, and the area of each adipocyte was measured using the Image J program. The average area of each adipocyte is also presented as graphs. Five to six mice per group were used for the preparation of H&E stained tissues; the area of adipocytes was measured in duplicate for each slide. Data represent the mean ± SD. * *p* < 0.05 compared to the No-treated group. # *p* < 0.05 compared to the HFD + Vehicle-treated group. Abbreviations: HFD, high-fat diet; LLEP, low concentration of LEP; HLEP, high concentration of LEP.

We further investigated whether LEP treatment for 16 weeks inhibits fat accumulation in abdominal fat tissue. To achieve this, the weight of abdominal fat and the average area of each adipocyte were measured in the HFD + LEP-treated mice. The decreased weight gains of abdominal fat in the HFD feeding group was not observed in the LLEP or HLEP-treated groups (Figure 2A). However, a remarkable decrease was detected in the average area of each adipocyte in hematoxylin and eosin (H&E) stained fat tissue. These levels were significantly and dose-dependently decreased in the HFD + LLEP- and HFD + HLEP-treated groups, as compared to the HFD + Vehicle-treated group (Figure 2B). These results indicate that LEP treatment inhibits the accumulation of abdominal fat tissue in HFD-induced obesity mice.

### 2.4. Inhibitory Effect of LEP on Hepatic Steatosis in Liver Tissue

**Figure 3:**
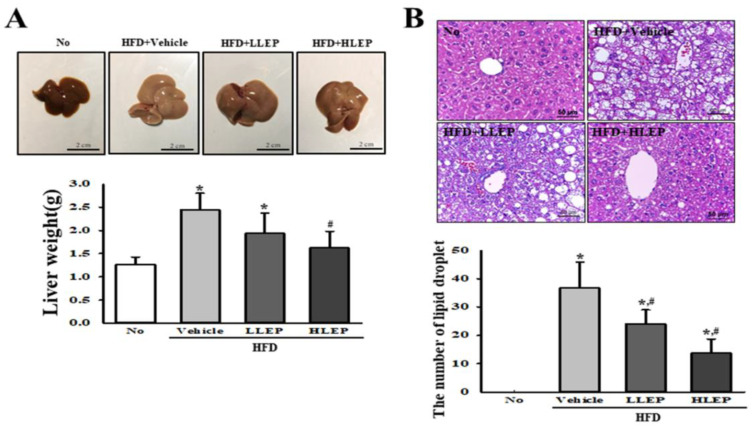
Measurement of liver weight and lipid droplet number in liver slide sections. (**A**) Livers collected from HFD-induced obese C57BL/6N mice, the subsequent to treatment with No, Vehicle, LLEP, or HLEP, were weighed using an electrical balance. Three to five mice per group were used for collecting livers, and their weight was measured in duplicate for each sample. (**B**) Lipid droplets were detected in H&E stained fat sections at 200× magnification, and the total number of lipid droplets was measured using the Leica Application Suite. Five to six mice per group were used for the preparation of H&E stained tissues, and the number of lipid droplets was counted in duplicate for each slide. Data represent the mean ± SD. * *p* < 0.05 compared to the No-treated group. # *p* < 0.05 compared to the HFD + Vehicle-treated group. Abbreviations: HFD, high-fat diet; LLEP, low concentration of LEP; HLEP, high concentration of LEP.

To investigate whether LEP treatment for 16 weeks inhibits hepatic steatosis in liver tissue, the weight and fat accumulation of liver were measured in the HFD-induced obesity model after LEP consumption. The weight of the liver was observed to be heavier in the HFD feeding group than those in the No treated group. However, these levels were not decreased in the HFD + LLEP- and HFD + HLEP-treated groups, as compared to the HFD + Vehicle-treated group (Figure 3A). A significant decrease pattern was observed in the average number of lipid droplets in liver tissue. Liver sections of the HFD + Vehicle-treated group showed dramatic increase in the number of lipid droplets compared to the No-treated group. In comparison, the number of lipid drops was decreased in the HFD + LLEP- and HFD + HLEP-treated groups (Figure 3B). These results indicate the capability of LEP to inhibit hepatic steatosis of liver tissue in HFD-induced obesity mice.

### 2.5. Effect of LEP on Adipogenesis and Lipogenesis of Liver Tissue during Anti-Hepatic Steatosis Effects

**Figure 4:**
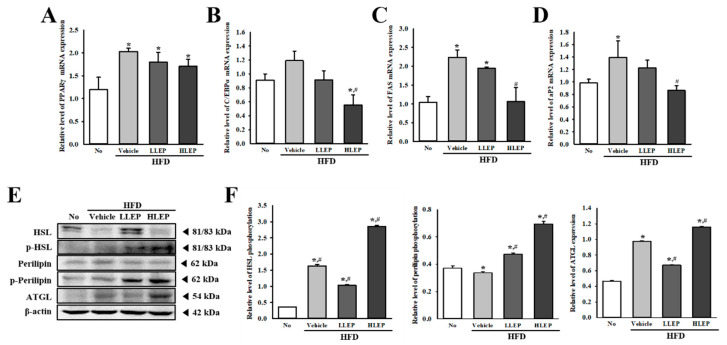
Expressions of lipogenesis associated genes and lipolysis associated proteins in liver tissue. HFD-induced obesity C57BL/6N mice were treated with No, Vehicle, LLEP, or HLEP for 16 weeks. RT-qPCR using specific primers was performed to analyze the expression levels of genes associated with (**A**,**B**) adipogenesis, and (**C**,**D**) lipogenesis in the liver. Three to five mice per group were used in the preparation of total RNA, and RT-qPCR were assayed in duplicate for each sample. (**E**,**F**) Western blot analysis was performed to detect the phosphorylation or expression of several lipolysis-associated proteins, including perilipin, p-perilipin, hormone-sensitive lipase (HSL), p-HSL, and adipose triglyceride lipase (ATGL). The intensity of each band was determined using an imaging densitometer, and the relative levels of the four proteins were calculated based on the intensity of actin. Three to five mice per group were used in the preparation of liver tissue homogenate, and Western blot analyses were assayed in duplicate for each sample. Data represent the mean ± SD. * *p* < 0.05 compared to the No-treated group. # *p* < 0.05 compared to the HFD + Vehicle-treated group. Abbreviations: HFD, high-fat diet; LLEP, low concentration of LEP; HLEP, high concentration of LEP; RT-qPCR, reverse transcription-quantitative polymerase chain reaction; HSL, hormone-sensitive lipase; ATGL, adipose triglyceride lipase.

To investigate whether anti-hepatic steatosis effects of LEP are accompanied by the inhibition of adipogenesis and lipogenesis, the mRNA levels of several key genes affecting adipogenesis, and lipogenesis were measured in the liver tissue using reverse transcription-quantitative polymerase chain reaction (RT-qPCR). Changes similar to the inhibition of hepatic steatosis were observed in the mRNA expressions of four adipogenesis and lipogenesis related genes (PPAR_γ_, C/EBPα, FAS, and aP2) in the HFD + Vehicle- and HFD + LEP-treated groups. The mRNA levels of these genes were higher in the HFD + Vehicle-treated group than the No-treated group. However, LEP treatment induced a dose-dependent decrease of PPAR_γ_, C/EBPα, FAS, and aP2 mRNA expressions, although the decrease rates were varied (Figure 4A–D). These results indicate that the anti-hepatic steatosis effect of LEP may be associated with the inhibition of adipogenesis and lipogenesis through the suppression of related gene expressions.

### 2.6. Effect of LEP on Lipolysis during Anti-Hepatic Steatosis Effects

We further examined the molecular mechanism for the lipolytic activity of LEP to investigate whether the anti-hepatic steatosis effects of LEP induce the activation of lipolysis. To achieve this, the expression and activation of key enzymes involved in TG metabolism were analyzed in the liver tissue of HFD + LEP-treated mice. As shown in Figure 4E,F, the phosphorylation and expression level of three major enzymatic proteins (perilipin, adipose triglyceride lipase (ATGL) and hormone-sensitive lipase (HSL)) were significantly enhanced in the HFD + HLEP-treated group, as compared to the HFD + Vehicle-treated group. However, these increase patterns were not clearly observed in the HFD + LLEP-treated group. Our data indicate that the anti-hepatic steatosis effect of LEP may be associated with the activation of lipolysis.

### 2.7. Inhibition Effect of LEP on HFD-Induced ER Stress

**Figure 5:**
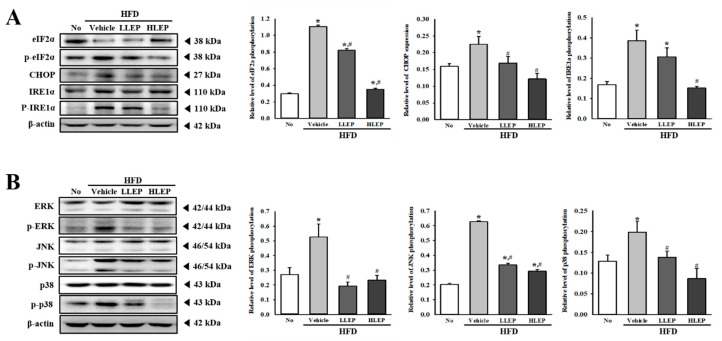
Expressions of key marker proteins for ER stress and MAP kinase signaling pathway in liver tissue. (**A**) The expression levels of ER stress markers, including eIF2α, p-eIF2α, CHOP, p-IRE1α and IRE1α, were detected in total liver proteins using specific antibodies. (**B**) Using specific antibodies, the expression levels of ERK, p-ERK, JNK, p-JNK, p38, and p-p38 in the MAPK signaling pathway were detected in total liver proteins. The band intensity of proteins was determined using an imaging densitometer, and the level of each protein was calculated based on the intensity of actin protein as an endogenous control. After then, the phosphorylation level of specific protein was calculated by dividing the level of phosphorylated proteins by the level of total proteins. Three to five mice per group were used in the preparation of tissue homogenate, and Western blot analysis was assayed in duplicate for each sample. Data represent the mean ± SD. * *p* < 0.05 compared to the No-treated group. # *p* < 0.05 compared to the HFD + Vehicle-treated group. Abbreviations: HFD, high-fat diet; LLEP, low concentration of LEP; HLEP, high concentration of LEP; ER, Endoplasmic reticulum; MAP; mitogen-activated protein; CHOP, C/EBP homologous protein, eIF2α, eukaryotic translation initiation factor 2α; IRE1α, inositol-requiring protein 1α.

To investigate whether anti-hepatic steatosis effects of LEP subsequently abolish ER stress, an alteration in the activation of key proteins during the ER stress response was evaluated in the liver of HFD-induced obesity mice treated with LEP. The changes in the phosphorylation and expression levels of three key proteins were very similar in all groups. The phosphorylation levels of eIF2α and IRE1α were remarkably enhanced after HFD administration, as compared to the No-treated group. However, these levels were dose-dependently inhibited in the HFD + LLEP and HFD + HLEP-treated groups (Figure 5A). A similar decrease was observed for CHOP expression, although the decrease rate differed (Figure 5A). Furthermore, the inhibitory effects of LEP in HFD-induced ER stress were evaluated for the involvement of the MAP kinase pathway. Enhanced levels of ERK, JNK, and p38 phosphorylation were variably recovered in the HFD + LLEP- and HFD + HLEP-treated groups (Figure 5B). Taken together, the above results indicate that the anti-hepatic steatosis activity of LEP successfully abolishes the HFD-induced ER stress through the regulation of the MAP kinase signaling pathway.

### 2.8. Inhibition Effects of LEP on ER Stress-Mediated Autophagy during Anti-Hepatic Steatosis Effects

**Figure 6:**
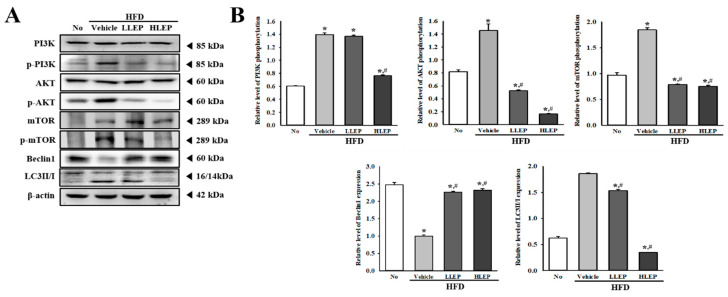
Expressions of key marker proteins for PI3K/AKT/mTOR pathway and autophagy in liver tissue. (**A**) Western blot image of marker proteins. The expression levels of PI3K, p-PI3K, AKT, p-AKT, mTOR, p-mTOR, Beclin1, and LC3II/I in total liver proteins were detected with specific antibodies. The level of β-actin is shown as the endogenous control. (**B**) Relative level of phosphorylation and expression of marker proteins. The band intensity of proteins was determined using an imaging densitometer, and the relative level of each protein was calculated based on the intensity of actin protein as an endogenous control. Three to five mice per group were used in the preparation of tissue homogenate, and Western blot analysis was assayed in duplicate for each sample. Data represent the mean ± SD. * *p* < 0.05 compared to the No-treated group. # *p* < 0.05 compared to the HFD + Vehicle-treated group. Abbreviations: HFD, high-fat diet; LLEP, low concentration of LEP; HLEP, high concentration of LEP; PI3k, phosphatidylinositol-3-kinase; mTOR, mammalian target of rapamycin.

The inhibitory role of LEP on autophagy in the sequential suppression of ER stress was examined by administering LEP to the HFD-induced obesity model. Alterations in the levels of markers for PI3K/AKT/mTOR signaling pathway and autophagy were detected in the liver tissue of HFD + LEP treated mice, with significant alterations being detected in the PI3K/AKT/mTOR signaling pathway. The phosphorylation levels of PI3K, AKT, and mTOR were higher in the HFD + Vehicle-treated group than the No-treated group. However, these levels remarkably decreased in the HFD + LEP-treated group when compared to the HFD + Vehicle-treated group (Figure 6A,B). We further examined the expression levels of Beclin1 and LC3 in the liver tissue, to investigate whether inhibition of the PI3K/AKT/mTOR signaling pathway after LEP treatment results in the recovery of autophagy. The levels of Beclin1 and LC3 proteins were significantly recovered in the HFD + LEP-treated group compared with the HFD + Vehicle- treated mice, although the recovery rates differed (Figure 6A,B). These results indicate that inhibition of ER stress induced by LEP treatment is associated with the regulation of the autophagy machinery, including the PI3K/AKT/mTOR signaling pathway and LC3 proteins.

### 2.9. Inhibitory Effects of LEP on Autophagy-Related Gene Transcriptions during Anti-Hepatic Steatosis

**Figure 7:**
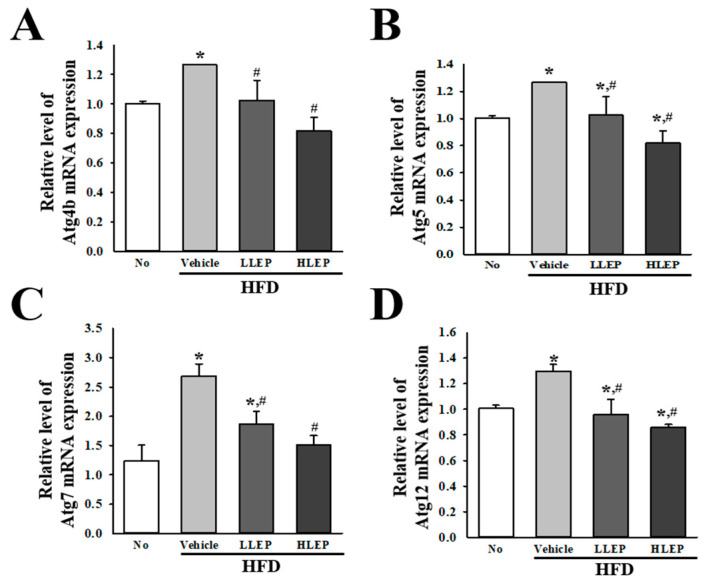
Transcription of autophagy-related genes in liver tissue. HFD-induced obesity C57BL/6N mice were treated with No, Vehicle, LLEP, or HLEP for 16 weeks. After purification of total RNA, the levels of (**A**) Atg4b, (**B**) Atg5, (**C**) Atg7, and (**D**) Atg12 mRNA in the total transcripts of liver tissue were measured by RT-qPCR analyses using their specific primers. The relative levels of the Atg4b, Atg5, Atg7, and Atg12 mRNA were calculated based on the intensity of β-actin as an endogenous control. Three to five mice per group were used in the preparation of the total RNAs, and RT-qPCR analysis was assayed in duplicate for each sample. Data represent the mean ± SD. * *p* < 0.05 compared to the No-treated group. # *p* < 0.05 compared to the HFD + Vehicle-treated group. Abbreviations: HFD, high-fat diet; RT-qPCR, reverse transcription-quantitative polymerase chain reaction; LLEP, low concentration of LEP; HLEP, high concentration of LEP.

Finally, we investigated whether recovery of autophagy and PI3K/AKT/mTOR signaling pathway induces the inhibition of autophagy-related gene transcriptions. To achieve this, alterations in the mRNA expression levels of four autophagy-related genes (Autophagy-related proteins (Atg) 4b, Atg5, Atg7, and Atg12) were evaluated in the total liver RNA of HFD + LEP-treated mice. Under conditions of hepatic steatosis, transcription levels of all genes were increased in the HFD + Vehicle-treated group as compared to the No-treated group. However, these levels were significantly and dose-dependently decreased after LEP administration, although the rates were varied for each gene (Figure 7). These results indicate that the LEP-induced inhibition of autophagy may be accompanied by transcriptional regulation of the autophagy-related genes.

## 3. Discussion

The beneficial effects of perilla oil have been investigated for the regulation and treatment of obesity, although these studies were not focused on the mechanism of ER stress-mediated autophagy. Most previous studies limitedly analyzed the suppressive effects of perilla oil alleviating the body weight, organ weight, lipid profile, and fat accumulation in specific organs after treatment for 12–18 weeks [25,26,29]. Also, a specific type of perilla oil extracted using the cold-pressed method was only considered to be a Western-style diet in a single [27], with no further follow-up study. The current study investigates the molecular mechanism of ER stress-mediated autophagy based on the inhibitory effect exerted by LEP for hepatic steatosis. The present results provide the first evidence of the molecular mechanism for ER stress-mediated autophagy, where the anti-hepatic steatosis effects of LEP were determined to be associated with regulation of ER stress and autophagy in the HFD-induced obesity model.

Plant essential oils are commonly isolated from various plant segments such as leaves, fruit, bark, root, wood, berries, seeds, flowers, and buds [30]. They contain numerous bioactive components, including free fatty acids, triglycerides, glycerol, phenolic compounds, and tocopherols [31]. Considering the variety of plant oils extracted, most consist of several fatty acids including α-linolenic acid (60.93%), oleic acid (16.21%), linoleic acid (14.72%), palmitic acid (5.94%), stearic acid (2.20%), arachidic acid (0.2%), palmitoleic acid (0.12%), behenic acid (0.03%), and lignoceric acid (0.01) [32]. The perilla oil largely used in previous studies for anti-obesity effects contains 58% α-linolenic acid [25]. In the present study, a similar level of fatty acids was founded in LEP, although the amounts of α-linolenic and linoleic acid were slightly higher (65.03%) and lower (12.11%), respectively. These differences could probably be attributed to the process of oil extraction and the quality of raw materials used; our study applied the cold-pressed method and used organic *P. frutescens* seed samples.

Alterations in bodyweight gain and serum lipid profiles are important indicators to establish the efficacy of anti-obesity therapeutic drugs and related products [33]. These alterations have also been observed in the HFD-induced obesity model treated with various essential oils. The treatment of garlic essential oil, citronella oil, ginger essential oil, black currant seed oil, and sweet orange essential oil are reported to induce a decrease in the bodyweight gain, as well as a decrease in the TG and TC levels in the HFD-induced obesity model [34,35,36,37,38]. Similar results were observed in the lipid profile of animals treated with perilla oil. Serum levels of TC, TG, and LDL-C were significantly decreased in the HFD-induced model after treatment with perilla oil. However, all mice treated with perilla oil and related diet remained their weight as a constant level [25,26,27,28]. In our study, although no significant change was detected in the bodyweight of HFD + LEP-treated mice, the TC, TG and LDL-C levels were decreased after LEP administration. Our results are very similar to previously reported results that evaluated the anti-obesity effects of perilla oil in the HFD-treated model. However, further research using essential oils of other plants and perilla oil is required to determine the difference in efficacy on weight loss.

Our study results indicate that LEP treatment for 16 weeks ameliorates the HFD-induced hepatic steatosis in C57BL/6N mice. The number of lipid droplets strongly decreased in the liver of HFD + LLEP- and HFD + HLEP-treated groups (Figure 3). These results are consistent with previous studies that report the reduction of HFD-hepatic steatosis after treatment with perilla oil. Histopathological analyses of liver tissue have revealed that the perilla oil-rich diet and Western-style diet containing 5.5% perilla oil reduce hepatic steatosis in the HFD-treated Sprague Dawley (SD) rats [26,27]. Moreover, 200 mg/kg or 1000 mg/kg/day of perilla oil decreased the hepatic lipid accumulation and improved the balance of lipogenic and lipolytic protein [28]. However, it is difficult to directly compare the degree of anti-hepatic steatosis effects between studies since the analysis methods differed in each study.

ER stress is induced when unfolded and misfolded proteins excessively accumulate in the ER lumen, or when calcium levels increase in the ER exhaust [39]. Studies in several animal models have reported that ER stress is tightly related to hepatic steatosis. Direct enhancement of ER stress induced with chemical agents and genetic mutations of ER stress molecules (including PERK/eIF2α, IRE1/ X-box binding protein 1 (XBP1) and ATF6) stimulate hepatic steatosis via controlling lipid synthesis and the inflammatory response [7,40,41]. Conversely, the pathogenesis of hepatic steatosis and obesity induces the ER stress response. Especially, HFD treatment induces the development of hepatic steatosis, type 2 diabetes, and insulin resistance, as well as increases the expressions of ER stress markers in liver tissue [42,43,44]. Therefore, the relationship between ER stress and hepatic steatosis is bilateral, since steatosis promotes ER stress, and the ER stress response leads to steatosis [45]. However, previous studies have not investigated whether the anti-obesity effect of plant essential oils is associated with ER stress. Therefore, in the current study, alteration of ER stress markers was analyzed in the liver tissue of HFD + LEP-treated mice, to investigate the mechanism and role of LEP during the anti-hepatic steatosis. Our results provide the first evidence that the anti-hepatic steatosis effects of LEP may tightly be linked to the regulation of eIF2α, IRE1α, and CHOP associated with the ER stress responses.

Finally, ER stress-mediated autophagy is tightly controlled to maintain homeostasis in the liver tissue of obese mice, although there is a lack of scientific evidence. In the liver of lean type mice, impairment of autophagy increases the ER stress, whereas rescuing induces the downregulation of ER stress markers such as LC3, Beclin1, Atg5, and Atg7 in the ob/ob and HFD-induced model [42]. Also, the levels of LC3, LC3-II, and p62 in autophagosome were reported to be enhanced in the non-alcoholic steatohepatitis (NASH) murine model, but patients with NASH show an increase of only p62 level [42,46,47,48]. A significant increase of several autophagy markers has been reported in the liver tissue of various animal models, including ob/ob mice [49], the HFD-treated C57BL/6J mice [50], and HFD-treated Wistar rats [51]. Furthermore, these enhanced marker levels were significantly reduced in the hepatic steatosis animal models after treatment with natural products, including grape seed proanthocyanidins [52], extracts of *Abelmoschus manihot* [28], Jiang Zhi [53], and epigallocatechin-3-gallate [54]; however, there was no study applying vegetable oil. Based on the above-associated studies, the present study especially focused on the applicability of this target in anti-hepatic steatosis activity of LEP. Our results provide the first evidence that LEP with anti-hepatic steatosis effects inhibits the ER stress-mediated autophagy in HFD-induced obesity models through the regulation of the PI3K/AKT/mTOR pathway. These results are in full agreement with previous results derived from the HFD-induced model after the administration of anti-autophagy products, although the treated products and analysis markers are different.

## 4. Materials and Methods

### 4.1. Extraction and Composition Analyses of LEP

Seed samples of *Perilla frutescens* were collected in October 2017 from plantations in Myrang City (Korea), and were characterized by Dr. Chung Yeoul Lee, Research Director at Gangrim Organics Co. (Miryang, Korea). Voucher specimens (WPC-18-001) were deposited in the Functional Materials Bank at the Pusan National University-Wellbeing Regional Innovation System (RIS) Center. Briefly, seeds of *P. frutescens* were washed with tap water and subsequently dried in a hot-air drying machine (JSR, Seoul, Korea) for 24 h at 60 °C. LEP was produced using a cold-press machine (Pungjin Food Machin Co., Mokpo, Korea) at 60 MPa, followed by filtration through a 6–8 µm membrane filter. The yield of LEP was determined to be 25–30 mL/100 g of seed samples. The composition and calorie contents of LEP were analyzed on an FID-equipped Gas Chromatograph (Agilent Technologies, Santa Clara, CA, USA) at the Traditional Microorganism Resources Center of Keimyung University. The calorie of LEP was determined to be 829 Kcal/100 mL.

### 4.2. Design of Animal Experiment

Eight-week-old C57BL/6N male mice were purchased from Samtako BioKorea Inc. (Osan, Korea), and provided *ad libitum* access to water and a standard irradiated chow diet (Samtako Bio-Korea Inc.). During the experiment, mice were maintained in specific pathogen-free (SPF) conditions under a strict light cycle (lights on at 08:00 h and off at 20:00 h) at 23 ± 2 °C and 50 ± 10% relative humidity. All C57BL/6N mice were handled at the Pusan National University-Laboratory Animal Resources Center, accredited by the Korea Food and Drug Administration (KFDA) (Accredited Unit Number-000231), and AAALAC International (Accredited Unit Number; 001525).

The Institutional Animal Care and Use Committee-Pusan National University (IACUC-PNU) approved the protocol for the animal experiment (PNU-2018-2011). All mice were acclimatized on a normal diet (D12450K; Research Diets, New Brunswick, NJ, USA) for 1 week. The C57BL/6N mice were then divided into four experimental groups (seven mice/group): (1) control group fed a normal diet (No-treated group), (2) group fed HFD plus Vehicle (Corn oil 10 mL/kg) (HFD + Vehicle-treated group), (3) group fed HFD plus 50% diluted LEP in corn oil 10 mL/kg (HFD + LLEP-treated group), and (4) group fed HFD plus original LEP of 10 mL/kg (HFD + HLEP-treated group). Mice of all HFD treatment groups consumed HFD containing 60% kcal fat purchased from Research Diets (#D12492, Research Diets, New Brunswick, USA) for 16 weeks. After 24 h of the final LEP treatment, all mice were euthanized using CO_2_ gas. The tissue samples and blood serum were subsequently acquired and stored in Eppendorf tubes at −70 °C, until assay.

### 4.3. Measurement of Body, Liver, and Abdominal Fat Weight

Throughout the experimental period, the bodyweight of mice treated with Vehicle or LEP was measured daily at 10:00 a.m. using an electronic balance (#AD-2.5 Mettler Toledo, Greifensee, Switzerland), according to the KFDA guidelines. In addition, the weights of liver and abdominal fat collected from the sacrificed C57BL/6N mice were determined using the same method employed to measure the body weight.

### 4.4. Serum Biochemical Analysis

After 16 weeks of feeding HFD diets and administrating LEP, whole blood was collected from the abdominal veins of all C57BL/6N mice after 18 h fasting. The blood samples were incubated for 30 min at room temperature in serum separating tubes (BD container, Franklin Lakes, NJ, USA), and serum was obtained by centrifugation at 1500× *g* for 15 min. Serum GLU, TC, TG, HDL-C, and LDL-C concentrations were analyzed by the automatic chemical analyzer (BS-120 Chemistry Analyzer, Mindray, China). All assays were conducted in duplicate using fresh serum.

### 4.5. Histopathological Analysis

Liver and fat tissues collected from mice of all subset groups were fixed overnight in 10% neutral buffered formaldehyde (pH 6.8). The dehydrated liver tissue was then embedded in paraffin wax. Next, a series of liver and fat sections (4 μm) were cut from the paraffin-embedded tissues using a Leica microtome (#DM500, Leica Microsystems, Bannockburn, IL, USA). These sections were collected on glass slides, deparaffinized with xylene (#8587-4410, DAEJUNG, Gyeonggi-do, Korea), rehydrated with graded ethanol (decreasing concentrations of 100–70%), and finally washed with distilled water. The liver and fat section slides were stained with hematoxylin (#MHS16, Sigma-Aldrich, St. Louis, MO, USA) and eosin (#HT110332, Sigma-Aldrich), washed with dH_2_O, and the number of lipid droplets was counted under the Leica Application Suite (Leica Microsystems, Heerbrugg, Switzerland). Also, the area occupied by adipocytes in the fat section was measured using the Image J program 1.52a (NIH, Bethesda, ML, USA).

### 4.6. RT-qPCR Analysis

Frozen liver tissue was chopped with scissors and homogenized in RNA Bee solution (#CS-105B, Tet-Test, Friendswood, TX, USA). Total RNA molecules were isolated by centrifugation at 15,000 rpm for 15 min, after which the concentration was measured by Nano Drop Spectrophotometers (Allsheng, Hangzhou, China). To examine the expression of each genes, total RNA (5 μg) from liver tissue was annealed with 500 ng of oligo-dT primer (#18418–012, Thermo Fisher Scientific, Wilmington, MA, USA) at 70 °C for 10 min. The complementary DNA (cDNA) was synthesized by Invitrogen Superscript II reverse transcriptase (#4376600, Thermo Fisher Scientific). qPCR was performed with the cDNA template (2 μL) and 2× Power SYBR Green (6 μL; Toyobo Life Science, Osaka, Japan) containing specific primers. The primer sequences for target gene expression identification used were presented in Table 2. qPCR was performed for 40 cycles using the following sequence: denaturation at 95 °C for 15 sec, followed by annealing and extension at 70 °C for 60 sec. Fluorescence intensity was measured at the end of the extension phase of each cycle. Threshold value for the fluorescence intensities of all samples was set manually. The reaction cycle at which the PCR products exceeded this fluorescence intensity threshold during the exponential phase of PCR amplification was considered, as the threshold cycle (Ct). Expression of the target gene was quantified relative to that of the housekeeping gene β-actin, based on a comparison of the Cts at constant fluorescence intensity, as per the Livak and Schmittgen’s method [55].

### 4.7. Western Blot Analysis

Liver tissue (50 mg) collected from each mice of subset groups was homogenized using the PRO-PREPTM Solution (#170841, iNtRON Biotechnology, Sungnam, Korea), and total protein extracts were collected by centrifugation at 13,000 rpm for 5 min. Protein concentrations were determined with the Pierce™ Bicinchonic Acid (BCA) Protein Assay Kit (Thermo Fisher Scientific). The prepared proteins (30 μg) were subsequently subjected to 10% sodium dodecyl sulfate-polyacrylamide gel electrophoresis (SDS-PAGE) for 2 h at 100 V, and transferred to a nitrocellulose membrane (GE Healthcare, Little Chalfont, UK) for 2 h at 40 V in transfer buffer (25 mM Trizma-base, 192 mM glycine, and 20% methanol). Membranes were then exposed to appropriate dilutions of primary antibodies and allowed to hybridize overnight at 4 °C; following antibodies were used: anti-HSL (#4107S, Cell Signaling Technology, Danvers, MA, USA), anti-p-HSL (#4139S, Cell Signaling Technology), anti-perilipin (#9349S, Cell Signaling Technology), anti-p-perilipin (#9621S, Cell Signaling Technology), anti-ATGL (#2138S, Cell Signaling Technology), anti-Beclin1 (#3738s, Cell Signaling Technology), anti-LC3-I/II (#3868s, Cell Signaling Technology), anti-PI3K (#4292s, Cell Signaling Technology), anti-p-PI3K (#4228s, Cell Signaling Technology), anti-AKT (#9272s, Cell Signaling Technology), anti-p-AKT (#4058s, Cell Signaling Technology), anti-mTOR (#2972s, Cell Signaling Technology), anti-p-mTOR (#2971s, Cell Signaling Technology), anti-JNK (#9252s, Cell signaling Technology), anti-p-JNK (#9251s, Cell signaling Technology), anti-ERK1/2 (#9102s, Cell signaling Technology), anti-p-ERK1/2 (#sc-7383, Santa Cruz Biotechnology, Dallas, TX, USA), anti-p38 (#9212s, Cell Signaling Technology), anti-p-p38 (#9211s, Cell Signaling Technology), anti-eIF2α (#9722S, Cell Signaling Technology), anti-p-eIF2α (#9721S, Cell Signaling Technology), anti-CHOP (#2895S, Cell Signaling Technology), anti-IRE1α (#NB100-2324, Novus Biologicals, Littleton, CO, USA), anti-p-IRE1α (#NB100-2323, Novus Biologicals), and anti-β-actin (#4967S, Cell Signaling Technology). The membranes were washed three times in a 10 mM Trizma-base (150 mM NaCl and 0.05% Tween-20) solution for 10 min upon removal primary antibodies solution and subsequently incubated with horseradish peroxidase-conjugated secondary antibody for 1 h at room temperature, after which they were washed again as described above, and developed using an enhanced chemiluminescence reagent plus kit (#DG-WF100, Dogen, Seoul, Korea). Finally, the results were quantified using the Image Analyzer System (Fluorchem FC2, Alpha Innotech, CA, USA) and expressed as the fold increase over the control values.

### 4.8. Statistical Significance Analysis

One-way ANOVA (SPSS for Windows, Release 10.10, Standard Version, Chicago, IL, USA) was used to determine the variance and identify significant differences between the No-treated group and the HFD fed groups, as well as between the Vehicle and LEP-treated groups. All values are presented as the means ± standard deviation (SD). A *p*-value < 0.05 is considered significant.

## 5. Conclusions

Taken together, the results of the current study demonstrate that LEP exerts anti-hepatic steatosis activity by successfully suppressing adipogenesis and lipogenesis, as well as activating lipolysis in the liver tissue of HFD-induced obese mice. In addition, the anti-hepatic steatosis effects of LEP are accompanied by suppression of ER stress-mediated autophagy, including the MAPK and PI3K/AKT/mTOR signaling pathways. The regulatory effects of LEP on the ER stress response and autophagy during the suppression of hepatic steatosis indicates the potential of LEP as an anti-obesity drug that reduces fat accumulation.

## Figures and Tables

**Table 1:** Concentration of TC, TG, LDL-C, HDL-C, and GLU in serum.

Factors	No	Vehicle	HFD	HLEP
			LLEP	
TC (g/dL)	83.8 ± 6.0	190.7 ± 10.9 *	152.7 ± 34.0 *^,#^	145.3 ± 24.9 *^,#^
TG (mg/dL)	55.4 ± 10.1	61.7 ± 9.2	49.7 ± 14.9	49.5 ± 14.0
LDL-C (mg/dL)	9.3 ± 2.4	35.0 ± 6.7 *	32.6 ± 4.6 *	21.8 ± 5.0 *^,#^
HDL-C (mg/dL)	74.6 ± 5.2	141.2 ± 5.9 *	144.2 ± 13.3 *	148.5 ± 14.5 *
GLU (mg/dL)	254.8 ± 41.1	448.6 ± 77.3 *	415.25 ± 43.8 *	352.2 ± 68.3 ^#^

Five to six mice per group were used for the preparation of serum, and the biochemical analyses were assayed in duplicate for each sample. Data represent the mean ± SD. * *p* < 0.05 compared to the No-treated group. ^#^ *p* < 0.05 compared to the HFD + Vehicle-treated group. Abbreviations: HFD, high-fat diet; LLEP, low concentration of LEP; HLEP, high concentration of LEP.

**Table 2:** Primer sequences for RT-PCR analyses.

Primer Name	Sequence (from 5′ to 3′)	Product Size (bp)
**PPAR-γ**		
Forward Reverse	GAG TTC ATG CTT GTG AAG GAT GCA AGG CAT ACT CTG TGA TCT CTT GCA CG	528
**C/EBP** **α**		
Forward Reverse	GTG GAC AAG AAC AGC AAC GAG TAC GGA ATC TCC TAG TCC TGG CTT GC	363
**FAS**		
Forward Reverse	GAT CCT GGA ACG AGA ACA CGA TCT GG AGA CTG TGG AAC ACG GTG GTG GAA CC	285
**aP2**		
Forward Reverse	GAA CCT GGA AGC TTG TCT CCA GTG GAT GCT CTT CAC CTT CCT GTC GTC TGC	233
**Atg4b**		
Forward Reverse	CTA TGT GGA GAC GCT GAA GCA CTG TTT C CTC TCC AGT CTC TCT ACA TCA GAA GAG	423
**Atg5**		
Forward Reverse	CCA AGA GTC AGC TAT TTG ACG TCC AAG GAA GAG CTG AAC TTG	178
**Atg7**		
Forward Reverse	CCT TGC TCA AAC ACT ACA GTG TGC TAT GTG TCA CGT CTC TAG	216
**Atg12**		
Forward Reverse	CCA TCC AAG GAC TCA TTG AC TTG CAG TAA TGC AGG ACC AG	166
**β-actin**		
Forward Reverse	TGG AAT CCT GTG GCA TCC ATG AAA C TAA AAC GCA GCT CAG TAA CAG TCC G	349
